# Supplementary material for: Identification of pathogens in culture-negative infective endocarditis cases by metagenomic analysis
Source: Ann Clin Microbiol Antimicrob. 2018 Dec 20;17:43. doi: 10.1186/s12941-018-0294-5 (PMC6300891; doi:10.1186/s12941-018-0294-5)
Supplement: Supplementary file 1 — Additional file 1: Table S1. Baseline Characteristics and clinical diagnosis of seven IE patients. Table S2. Phenotype of the seven study patients. Table S3. Detail result for AMR detection for data from different platforms. Table S4. Information of sequencing data from Nanopore MinION platform with seven samples. Table S5. Stable pathogen detected time for different cutoff of detected reads number in seven samples’ nanopore sequence data. Table S6. Detail result for species identification of BGI data. Table S7. Detail result for species identification of nanopore data. Table S8. Comparision for host percentage between two difference platforms. [file 12941_2018_294_MOESM1_ESM.docx]

**Additional Tables**

**Table S1.** Baseline Characteristics and clinical diagnosis of seven IE patients.

| Characteristic | IE Patients  (N=7) |
| --- | --- |
| Mean (±SD) age-yr | 48.3±7.4 |
| Male sex no. (%) | 6 (85.7%) |
| LVEF (%) | 64.4±5.4 |
| Valve type no. (%) |  |
| AV | 5 (71.4%) |
| MV | 2 (28.6%) |
| Vegetation no. (%) |  |
| AV | 3 (42.9%) |
| MV | 2 (28.6%) |
| AV+MV | 1 (14.3%) |
| Not found | 1 (14.3%) |
| Antibiotics used before admission no. (%) | 7 (100%) |
| Laboratory data |  |
| CRP(mg/L) | 30.4±24.0 |
| ESR(mm/h) | 29.9±32.9 |

AV, aortic valve; MV, mitral valve; TV, tricuspid valve; CRP, C reactive protein; ESR, erythrocyte sedimentation rate, LVEF, Left ventricular ejection fractions.

**Table S2.** Phenotype of the seven study patients.

| Case No | Gender | Age | Valve type^a^ | AUBA^b^ | vegetation | CRP^c^ | ESR^d^ | LVEF  (%)^e^ |
| --- | --- | --- | --- | --- | --- | --- | --- | --- |
| 1 | m | 50 | AV | moxifloxacin | AV | 7.04 | 28 | 65 |
| 2 | m | 52 | AV | Chinese medcine | not found | 20.7 | 21 | 70 |
| 3 | m | 38 | MV | penicillin  vancomycin  amikacin | MV | 60.2 | 2 | 56 |
| 4 | f | 56 | AV | vancomycin | AV | 4.36 | 31 | 67 |
| 5 | m | 57 | AV | vancomycin | AV | 20.5 | 14 | 69 |
| 6 | m | 41 | AV | Vancomycin  penicillin | AV,TV | 36.2 | 12 | 58 |
| 7 | m | 44 | MV | linezolid  Cefperazone-Sulbactam. | MV | 63.9 | 101 | 66 |

^a^AV, aortic valve; MV, mitral valve; TV, tricuspid valve;

^b^AUBA, antibiotics used before admission;

^c^CRP, c reactive protein;

^d^ESR, erythrocyte sedimentation rate;

^e^LVEF, left ventricular ejection fraction;

**Table S3.** Detail result for AMR detection for data from different platforms.

| Platform | Sample ID | Card aro ID | Aro name | Number of alignments | Depth | Max evalue |
| --- | --- | --- | --- | --- | --- | --- |
| BGI | A2 | 3001301 | RlmA(II) | 23 | 3.13192 | 3.44E-31 |
|  |  | 3000025 | patB | 19 | 1.958869 | 2.19E-33 |
|  |  | 3000375 | ErmB | 16 | 2.903743 | 4.06E-54 |
|  |  | 3000186 | tetM | 32 | 2.048933 | 3.85E-36 |
|  | A5 | 3000186 | tetM | 93 | 5.7595 | 7.29E-31 |
|  |  | 3000025 | patB | 32 | 3.163668 | 8.10E-32 |
|  |  | 3001301 | RlmA(II) | 27 | 3.607774 | 8.41E-32 |
|  |  | 3000375 | ErmB | 38 | 6.872995 | 6.17E-45 |
|  |  | 3000822 | pmrA | 8 | 0.741667 | 8.86E-32 |
|  | A7 | 3000186 | tetM | 43 | 5.7595 | 7.29E-31 |
|  |  | 3000375 | ErmB | 11 | 6.872995 | 6.17E-45 |
| Nanopore | A5 | 3000375 | ErmB | 1 | 0.731283 | 0 |
|  | A7 | 3000186 | tetM | 4 | 1.79646 | 5.91E-96 |
|  |  | 3000192 | tetS | 3 | 1.361371 | 2.87E-73 |
|  |  | 3000190 | tetO | 1 | 0.716146 | 0 |
|  |  | 3000196 | tet32 | 1 | 0.69375 | 2.75E-134 |
|  |  | 3000556 | tet44 | 1 | 0.667879 | 5.69E-86 |
|  |  | 3000375 | ErmB | 4 | 3.235294 | 5.75E-149 |

**Table S4.** Information of sequencing data from Nanopore MinION platform with seven samples.

| Sample ID | Data size(bp) | Reads number | Reads median length(K) | Reads avgerage length(K) | Base QC median | Base QC average |
| --- | --- | --- | --- | --- | --- | --- |
| A1.1 | 128307097 | 167880 | 0.6 | 0.764 | 12 | 13.43 |
| A1.2 | 94999295 | 127644 | 0.6 | 0.744 | 16 | 17.86 |
| A2.1 | 223203393 | 153751 | 1 | 1.452 | 13 | 14.16 |
| A2.2 | 261951047 | 212366 | 0.9 | 1.233 | 16 | 17.6 |
| A3 | 363825276 | 166549 | 1.1 | 1.865 | 12 | 13.23 |
| A4 | 365589547 | 425865 | 1.6 | 0.858 | 14 | 12.94 |
| A5 | 605126379 | 540063 | 0.8 | 1.12 | 16 | 17.86 |
| A6 | 170578361 | 127634 | 0.8 | 1.336 | 12 | 13.39 |
| A7 | 313922260 | 196334 | 0.8 | 1.599 | 17 | 18.06 |

**Table S5.** Stable pathogen detected time for different cutoff of detected reads number in seven samples’ nanopore sequence data.

| Sample ID | Pathogen reads number | 2 Reads detected time (minutes) | 5 Reads detected time (minutes) | 10 Reads detected time (minutes) |
| --- | --- | --- | --- | --- |
| A1.1 | 24 | 6.52 | 60.58 | 91.03 |
| A1.2 | 16 | 23.58 | 41.83 | 162.2 |
| A2.1 | 13 | 33.65 | 196.81 | 530.29 |
| A2.2 | 25 | 51.3 | 86.65 | 166.78 |
| A3 | 68 | 14.28 | 19.5 | 30.71 |
| A4 | 2106 | 0.3 | 1.1 | 2.2 |
| A5 | 317 | 4.95 | 12.18 | 20.31 |
| A6 | 42 | 24.85 | 46.58 | 92.08 |
| A7 | 3379 | 0.36 | 0.85 | 1.65 |

**Table S6.** Detail result for species identification of BGI data.

| Sample ID | Genus name | Species  name | TaxID | Species  genome size | Support reads num | Unique support reads num | Query length | Relative abundance | Normalized relative abundance |
| --- | --- | --- | --- | --- | --- | --- | --- | --- | --- |
| A1 | Streptococcus | Streptococcus gordonii | 1302 | 2196662 | 4465 | 4260 | 487901 | 81.1 | 82.4 |
|  | Streptococcus | Streptococcus sanguinis | 1305 | 2388435 | 738 | 618 | 70738 | 11.76 | 10.99 |
|  | Streptococcus | Streptococcus cristatus | 45634 | 2142100 | 242 | 167 | 18590 | 3.09 | 3.22 |
|  | Streptococcus | Streptococcus oralis | 1303 | 3925600 | 207 | 105 | 11693 | 1.94 | 1.1 |
|  | Streptococcus | Streptococcus intermedius | 1338 | 1996214 | 69 | 38 | 4265 | 0.71 | 0.79 |
|  | Streptococcus | Streptococcus parasanguinis | 1318 | 2153652 | 50 | 35 | 4038 | 0.67 | 0.7 |
|  | Streptococcus | Streptococcus mitis | 28037 | 2146611 | 42 | 16 | 1636 | 0.27 | 0.28 |
|  | Streptococcus | Streptococcus pneumoniae | 1313 | 2038615 | 34 | 13 | 1528 | 0.25 | 0.28 |
|  | Streptococcus | Streptococcus massiliensis | 313439 | 1864153 | 17 | 11 | 1228 | 0.2 | 0.24 |
| A2 | Streptococcus | Streptococcus oralis | 1303 | 1958690 | 31754 | 25275 | 3270958 | 79.4 | 81.01 |
|  | Streptococcus | Streptococcus pneumoniae | 1313 | 2038615 | 3563 | 2501 | 324102 | 7.87 | 7.72 |
|  | Streptococcus | Streptococcus mitis | 28037 | 2146611 | 3372 | 2253 | 293183 | 7.12 | 6.63 |
|  | Streptococcus | Streptococcus gordonii | 1302 | 2196662 | 714 | 463 | 60569 | 1.47 | 1.34 |
|  | Streptococcus | Streptococcus cristatus | 45634 | 2142100 | 604 | 450 | 57920 | 1.41 | 1.32 |
|  | Streptococcus | Streptococcus parasanguinis | 1318 | 2153652 | 398 | 219 | 27904 | 0.68 | 0.63 |
|  | Streptococcus | Streptococcus sanguinis | 1305 | 2388435 | 252 | 170 | 21621 | 0.52 | 0.44 |
|  | Streptococcus | Streptococcus equinus | 1335 | 1930741 | 96 | 85 | 10980 | 0.27 | 0.28 |
|  | Streptococcus | Streptococcus anginosus | 1328 | 2233640 | 117 | 80 | 9921 | 0.24 | 0.21 |
|  | Streptococcus | Streptococcus massiliensis | 313439 | 1864153 | 98 | 72 | 9386 | 0.23 | 0.25 |
|  | Streptococcus | Streptococcus salivarius | 1304 | 2188923 | 103 | 66 | 8301 | 0.2 | 0.18 |
| A3 | Coxiella | Coxiella burnetii | 777 | 1995488 | 4014 | 3921 | 479673 | 100 | 100 |
| A4 | Bartonella | Bartonella quintana | 803 | 1581384 | 29676 | 29438 | 3560025 | 99.38 | 99.55 |
|  | Neisseria | Neisseria mucosa | 488 | 2169437 | 187 | 179 | 22301 | 0.62 | 0.45 |
| A5 | Streptococcus | Streptococcus oralis | 1303 | 1958690 | 68435 | 54881 | 6925459 | 80.38 | 81.74 |
|  | Streptococcus | Streptococcus pneumoniae | 1313 | 2038615 | 7082 | 4739 | 598634 | 6.95 | 6.79 |
|  | Streptococcus | Streptococcus mitis | 28037 | 2146611 | 7078 | 4698 | 591483 | 6.86 | 6.37 |
|  | Streptococcus | Streptococcus gordonii | 1302 | 2196662 | 1910 | 1511 | 191459 | 2.22 | 2.01 |
|  | Streptococcus | Streptococcus cristatus | 45634 | 2142100 | 1583 | 1146 | 143923 | 1.67 | 0.85 |
|  | Streptococcus | Streptococcus sanguinis | 1305 | 2388435 | 890 | 588 | 74642 | 0.87 | 0.40 |
|  | Streptococcus | Streptococcus parasanguinis | 1318 | 2153652 | 603 | 377 | 47609 | 0.55 | 0.51 |
|  | Streptococcus | Streptococcus thermophilus | 1308 | 1929905 | 292 | 208 | 25892 | 0.3 | 0.31 |
| A6 | Streptococcus | Streptococcus sanguinis | 1305 | 2388435 | 380 | 370 | 43020 | 87.4 | 86.2 |
|  | Streptococcus | Streptococcus gordonii | 1302 | 2196662 | 46 | 36 | 4019 | 8.16 | 8.76 |
|  | Streptococcus | Streptococcus cristatus | 45634 | 2142100 | 18 | 15 | 1683 | 3.42 | 3.76 |
|  | Streptococcus | Streptococcus massiliensis | 313439 | 1864153 | 7 | 6 | 501 | 1.02 | 1.29 |
| A7 | Streptococcus | Streptococcus anginosus | 1328 | 2233640 | 47829 | 45880 | 5453989 | 88.69 | 87.82 |
|  | Streptococcus | Streptococcus intermedius | 1338 | 1996214 | 5228 | 3770 | 448924 | 7.3 | 8.09 |
|  | Filifactor | Filifactor alocis | 143361 | 1931012 | 949 | 630 | 74433 | 1.21 | 1.39 |
|  | Streptococcus | Streptococcus massiliensis | 313439 | 1864153 | 517 | 277 | 32777 | 0.53 | 0.63 |
|  | Streptococcus | Streptococcus cristatus | 45634 | 2142100 | 373 | 230 | 27148 | 0.44 | 0.46 |
|  | Streptococcus | Streptococcus gallolyticus | 315405 | 2492900 | 334 | 217 | 26246 | 0.43 | 0.38 |
|  | Streptococcus | Streptococcus oralis | 1303 | 3925600 | 317 | 215 | 25777 | 0.42 | 0.24 |
|  | Streptococcus | Streptococcus mutans | 1309 | 2032925 | 236 | 202 | 24178 | 0.39 | 0.43 |
|  | Streptococcus | Streptococcus gordonii | 1302 | 2196662 | 262 | 155 | 18464 | 0.3 | 0.3 |
|  | Streptococcus | Streptococcus sanguinis | 1305 | 2388435 | 296 | 151 | 17820 | 0.29 | 0.27 |

**Table S7.** Detail result for species identification of nanopore data.

| Sample ID | Genus name | Species  name | TaxID | Species  genome size | Support reads num | Unique support reads num | Query length | Relative abundance | Normalized relative abundance |
| --- | --- | --- | --- | --- | --- | --- | --- | --- | --- |
| A1.1 | Streptococcus | Streptococcus gordonii | 1302 | 2196662 | 24 | 23 | 25269 | 100.00% | 100.00% |
| A1.2 | Streptococcus | Streptococcus gordonii | 1302 | 2196662 | 16 | 16 | 22003 | 100.00% | 100.00% |
| A2.1 | Streptococcus | Streptococcus oralis | 1303 | 1958690 | 13 | 13 | 22945 | 100.00% | 100.00% |
| A2.2 | Streptococcus | Streptococcus oralis | 1303 | 1958690 | 25 | 23 | 19502 | 100.00% | 100.00% |
| A3 | Coxiella | Coxiella burnetii | 777 | 1995488 | 68 | 68 | 67040 | 100.00% | 100.00% |
| A4 | Bartonella | Bartonella quintana | 803 | 1581384 | 2106 | 2081 | 3099223 | 100.00% | 100.00% |
| A5 | Streptococcus | Streptococcus oralis | 1303 | 1958690 | 317 | 302 | 601776 | 94.52% | 94.70% |
|  | Streptococcus | Streptococcus pneumoniae | 1313 | 2038615 | 24 | 20 | 34869 | 5.48% | 5.29% |
| A6 | Streptococcus | Streptococcus sanguinis | 1305 | 2388435 | 42 | 42 | 76221 | 100.00% | 100.00% |
| A7 | Streptococcus | Streptococcus anginosus | 1328 | 2233640 | 3379 | 3302 | 4221132 | 91.73% | 90.77% |
|  | Streptococcus | Streptococcus intermedius | 1338 | 1996214 | 303 | 246 | 274534 | 5.97% | 6.60% |
|  | Filifactor | Filifactor alocis | 143361 | 1931012 | 84 | 70 | 105758 | 2.30% | 2.63% |

**Table S8.** Comparision for host percentage between two difference platforms.

| Platform | Sample ID | Host data size (Mbp) | Host reads num | Host percentage | Nonhost data size (Mbp) | Nonhost reads num | Nonhost percentage |
| --- | --- | --- | --- | --- | --- | --- | --- |
| **BGI** | A1 | 1949 | 16658441 | 99.37% | 12 | 274192 | 0.63% |
|  | A2 | 8184 | 62576932 | 99.45% | 45 | 1066194 | 0.55% |
|  | A3 | 1622 | 12160178 | 99.31% | 11 | 256547 | 0.69% |
|  | A4 | 2109 | 16859269 | 98.98% | 21 | 474247 | 1.02% |
|  | A5 | 4279 | 33432672 | 99.32% | 29 | 484815 | 0.68% |
|  | A6 | 1467 | 12705622 | 98.40% | 23 | 641708 | 1.60% |
|  | A7 | 2872 | 23479079 | 98.87% | 32 | 638742 | 1.13% |
| **Nanopore** | A1.1 | 103 | 107899 | 90.28% | 11 | 12602 | 9.72% |
|  | A1.2 | 81 | 84342 | 98.96% | 0.852 | 964 | 1.04% |
|  | A2.1 | 217 | 123049 | 97.41% | 5 | 4418 | 2.59% |
|  | A2.2 | 250 | 167809 | 98.92% | 2 | 2426 | 1.08% |
|  | A3 | 310 | 133441 | 98.29% | 5 | 3811 | 1.87% |
|  | A4 | 306 | 267971 | 95.41% | 14 | 14322 | 1.71% |
|  | A5 | 576 | 411941 | 98.4% | 9 | 8015 | 4.59% |
|  | A6 | 157 | 86543 | 95.22% | 7 | 5925 | 4.78% |
|  | A7 | 295 | 132731 | 97.05% | 9 | 7122 | 2.95% |
