# Supplementary figures and images for: Identification of pathogens in culture-negative infective endocarditis cases by metagenomic analysis
Source: Ann Clin Microbiol Antimicrob. 2018 Dec 20;17:43. doi: 10.1186/s12941-018-0294-5 (PMC6300891; doi:10.1186/s12941-018-0294-5)

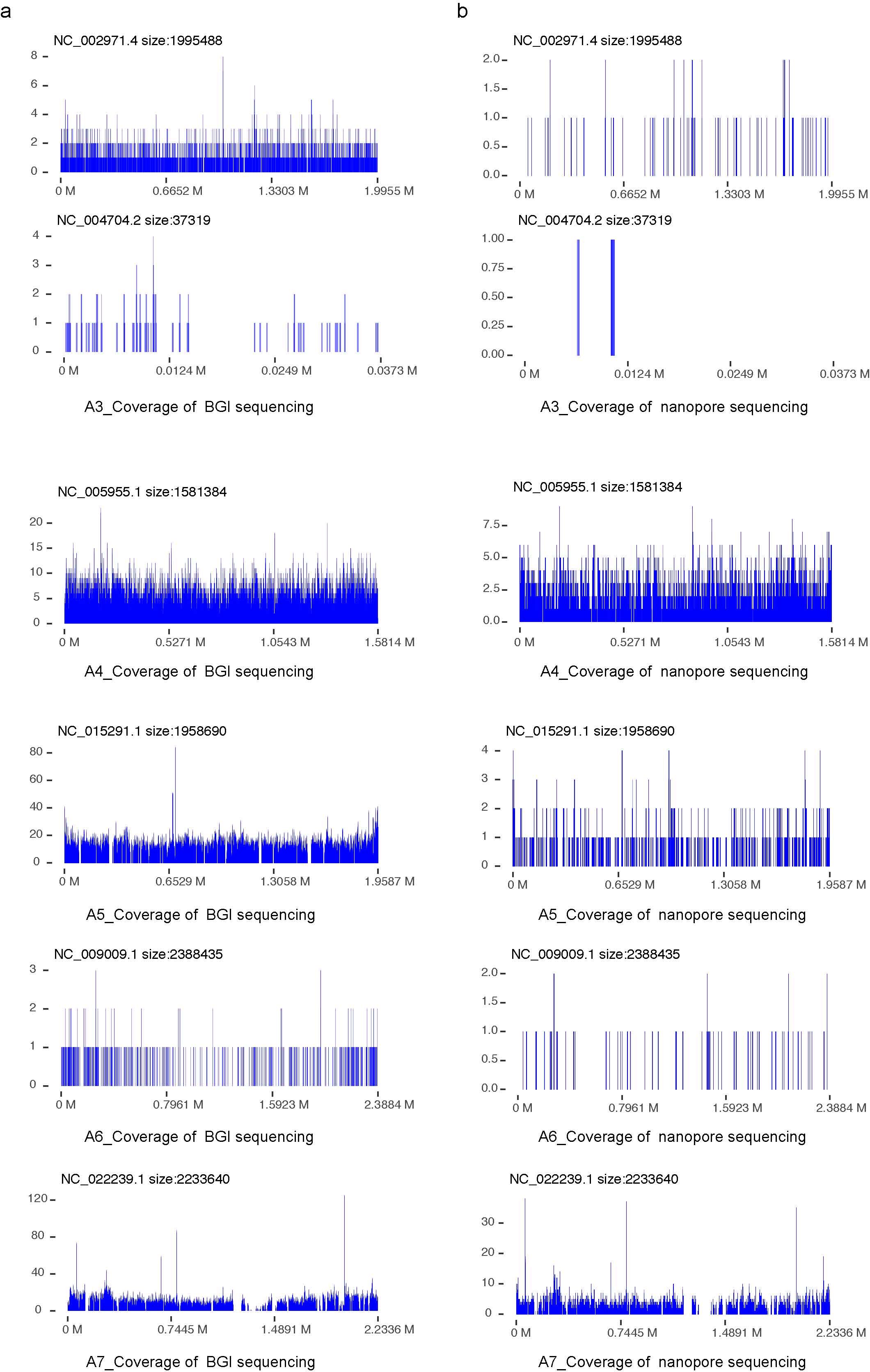

Supplement: Supplementary file 2 — Additional file 2: Fig. S1. Pathogen coverage of A3, A4, A5, A6 and A7 sequencing data with both NGS and Nanopore MinION platforms. a) the coverage density plot in detected pathogen genome for NGS sequence from BGI platform of A3 to A7 samples; b) the coverage density plot in detected pathogen genome for nanopore sequencing from MinION sequencer of A3 to A7 samples. The detected pathogens for A3 to A7 are Coxiella burnetii (NC_002971.4 and NC_004704.2), Bartonella Quintana (NC_005955.1), Streptococcus oralis (NC_015291.1), Streptococcus sanguinis (NC_009009.1) and Streptococcus anginosus (NC_022239.1). [file 12941_2018_294_MOESM2_ESM.jpg]
